# Community Next Steps for Making Globally Unique Identifiers Work for Biocollections Data

**DOI:** 10.3897/zookeys.494.9352

**Published:** 2015-04-06

**Authors:** Robert P. Guralnick, Nico Cellinese, John Deck, Richard L. Pyle, John Kunze, Lyubomir Penev, Ramona Walls, Gregor Hagedorn, Donat Agosti, John Wieczorek, Terry Catapano, Roderic D. M. Page

**Affiliations:** 1 Florida Museum of Natural History, University of Florida, Gainesville, FL 32611-2710 USA; 2 Berkeley Natural History Museums, University of California, Berkeley, California, USA; 3 Department of Natural Sciences, Bernice P. Bishop Museum, Honolulu, HI USA 96817; 4 California Digital Library, University of California Office of the President, Oakland, CA USA; 5 Institute of Biodiversity and Ecosystem Research, Bulgarian Academy of Sciences, and Pensoft Publishers, Sofia, Bulgaria; 6 iPlant Collaborative, University of Arizona,Tucson, AZ 85721; 7 Museum für Naturkunde, Leibniz-Institut für Evolutions- und Biodiversitätsforschung, Invalidenstraße 43, 10115 Berlin, Germany; 8 Plazi, Zinggstrasse 16, 3007 Bern, Switzerand; 9 Museum of Vertebrate Zoology, University of California, Berkeley, CA USA. United States of America. 94720-3160; 10 Institute of Biodiversity, Animal Health and Comparative Medicine, College of Medical, Veterinary and Life Sciences, University of Glasgow Glasgow, G12 8QQ. UK

**Keywords:** Biocollections, identifiers, Globally Unique Identifiers, GUIDs, field collections, legacy collections, linked open data, semantic publishing

## Abstract

Biodiversity data is being digitized and made available online at a rapidly increasing rate but current practices typically do not preserve linkages between these data, which impedes interoperation, provenance tracking, and assembly of larger datasets. For data associated with biocollections, the biodiversity community has long recognized that an essential part of establishing and preserving linkages is to apply globally unique identifiers at the point when data are generated in the field and to persist these identifiers downstream, but this is seldom implemented in practice. There has neither been coalescence towards one single identifier solution (as in some other domains), nor even a set of recommended best practices and standards to support multiple identifier schemes sharing consistent responses. In order to further progress towards a broader community consensus, a group of biocollections and informatics experts assembled in Stockholm in October 2014 to discuss community next steps to overcome current roadblocks. The workshop participants divided into four groups focusing on: identifier practice in current field biocollections; identifier application for legacy biocollections; identifiers as applied to biodiversity data records as they are published and made available in semantically marked-up publications; and cross-cutting identifier solutions that bridge across these domains. The main outcome was consensus on key issues, including recognition of differences between legacy and new biocollections processes, the need for identifier metadata profiles that can report information on identifier persistence missions, and the unambiguous indication of the type of object associated with the identifier. Current identifier characteristics are also summarized, and an overview of available schemes and practices is provided.

## Introduction

The current biodiversity and genomic fields are characterized by large and rapidly growing digital datasets. While this trend in digitizing the global biodiversity knowledge base is valuable and important for accessing and synthesizing biodiversity information in the era of the Internet and Big Data, much of this information remains only loosely integrated. Efforts to cross-link otherwise disconnected silos of data (Page 2008, 2009) still rely on largely imprecise points of intersection, such as text-string taxon names (as proxies for taxon concepts), combinations of institution codes, collection codes, and catalog numbers (as labels for biological specimens and other samples), and aggregates of metadata that allow inferring equivalency (e.g., a combination of place, time, and participants for collecting events).

The necessary solution to build more connected, cross-linked and digitially accessible Internet content is to assign recognizable, persistent, globally unique, stable identifiers to biocollections specimens and data objects. While effort has been put forth on applicability statements for both Life Science Identifiers (LSIDs) and globally unique identifiers (GUIDs) (Pereira et al. 2007, Richards 2009), and on other fronts (Pyle 2006, Cryer et al. 2009, Baskauf 2010, Richards et al. 2011, TDWG 2013, Bouchout Declaration 2014), no single solution or clear best practice has taken hold in the biocollections community. To illustrate, Table 1 shows some example of identifiers associated with data mobilised by GBIF and includes LSIDs, URNs, HTTP-URIs (URLs) of various types, and DOIs (See Box 1 for explanations of abbreviations used in this article). The community has also struggled to define its view on identifier and dereferencing service persistence, and whether physical objects and abstract concepts should have identifiers that include embedded information on dereferencing services and protocols (a dereferenceable identifier contains an Internet protocol that directs a client to information about the resource it identifies), or whether functions of object identification and dereferencing should be decoupled. Further, and perhaps most important, the next steps towards a community-wide GUID solution are unclear.

**Table 1.** Examples of identifiers in use for biological samples in the GBIF database.

GBIF occurrence	Identifier type	Identifier	Catalog number	Collection
872747863	LSID	urn:lsid:biosci.ohio-state.edu:osuc_occurrences:OSUC__169968	OSUC 169968	C.A. Triplehorn Insect Collection
896421698	URN	urn:occurrence:Arctos:MVZ:Bird:157675:1526959	MVZ 157675	MVZ Bird Collection
784060956	URN	urn:catalog:UMMZ:Mammals:171041	UMMZ 71041	UMMZ Mammal Collection
575336458	HTTP URI	http://data.rbge.org.uk/herb/E00115694	E00115694	Royal Botanic Garden Edinburgh Herbarium
1050474791	HTTP URI	http://arctos.database.museum/guid/UAM:Ento:230092	UAM 230092	UAM Entomology Collection
1050474791	DOI	10.7299/X7VQ32SJ	UAM 230092	UAM Entomology Collection
624211191	UUID	EF0A4D3E-702F-4882-81B8-CA737AEB7B28	UF 161444	UF FLMNH Ichthyology
476850316	Darwin Core Triplet	MCZ:Mamm:8831	MCZ 8831	Museum of Comparative Zoology, Harvard University

**Box 1.** Abbreviations and the full spelled out version or more detailed meaning.

ABCD	Access to Biological Collections Data
ARK	Archival Resource Key
BCO	Biological Collections Ontology
DMP	Data Management Plan
DOI	Digital Object Identifier
EZID	A type of identifier & system run by California Digital Library
GBIF	Global Biodiversity Information Facility
GRBio	Global Repository of Biorepositories
GUID	Globally Unique Identifier
HTTP-URI	HTTP Uniform Resource Identifier
IGSN	International Geosample Number
LOD	Linked Open Data
LSID	Life Sciences Identifier
NEON	National Ecological Observatory Network
OCR	Optical Character Recognition
TDWG	Biodiversity Information Standards
URI	Uniform Resource Identifier
URL	Uniform Resource Locator
URN	Uniform Resource Name
UUID	Universally Unique Identifier

The application of identifiers to biocollections and the physical (and conceptual) objects they contain is complicated by both long and ingrained identifier curation practice, and a rapidly changing technology landscape. Legacy collections often have a checkered past of provenance-tracking; as a result, essential linkages between data and collections have been lost due to lack of coordination and data practices predating digital recording. New, “born-digital” sampling methods promise to open floodgates of data and can make it easier to assign globally unique identifiers at the point of data creation. Thus, the optimal identifier solutions for new collections may be different than those for legacy data. Adding to the challenge, vast amounts of biodiversity data are in the scientific literature, which is the oldest form of biodiversity reporting. These data can be mined from the legacy literature but are largely “hidden” in non-semantic formats. In the future, advances in digital publishing will enable data to be more thoroughly linked to the literature, and vice-versa (Penev et al. 2010), thus laying the foundation for new best practices for citing datasets by means of identifiers.

In order to further progress on this critical issue, a group of biocollections and biodiversity informatics experts and stakeholders (Appendix 1) assembled at the Stockholm Museum of Natural History, 25–26 October 2014 to lay out a set of recommendations and next steps for community-wide approaches to globally unique identifier assignment, persistence, and dereferencing. After the opening discussions and compiling of key identifier characteristics (Box 2), the participants organized into four subgroups during the meeting: New biocollections, legacy biocollections, semantically enabled publications, and cross-cutting issues. In this paper we review the workshop results under those four headings and summarise consensus views on what should happen next.

**Box 2.** Below the main characteristics of identifier schemes are listed. The list is not meant to be exhaustive but is intended to cover the major differences across different approaches.

Identifier Schemes: support **locally unique** (e.g., catalog numbers) and/or **globally unique** (e.g. DOIs, URLs or UUIDs) identifiers. Global uniqueness is vital to minimize ambiguity; provide identifiers that are **actionable.** Actionable identifiers may rely on special knowledge (e.g. for LSIDs, DOIs, or http services for plain identifiers) or they may rely on Internet standards (URIs); may require resolvers to support access to the **object** and to its **metadata**; for example, **content negotiation** (e.g., used by Linked Open Data) supports the provision of a human-readable object in one context and machine-readable metadata (e.g., RDF, JSON) in another context; additionally, **inflections** (e.g., ARK) let an ordinary user add to the identifier to request the object or its metadata may use **centralized** (e.g., purl.org, doi.org, n2t.net) or **decentralized dereferencing hosts** (e.g., an institutional site); may support **transparent identifiers** (e.g., identifier strings that contain information which can lead to semantic guesses by humans, such as collection numbers, collectors’ initials, or institutional names) or **opaque identifiers**, e.g. strings of letters and digits created by software (counter, UUID generator, Noid minter); may come with **fees** for creation of an identifier (e.g. DOIs); may come with **fees** for the use of the resolver; these fees, which affect **scalability**, are separate from the time and effort required of end-providers no matter which identifier scheme they use (object curation, disk storage, updating resolver data as the object moves, etc.); may come with **metadata** requirements (e.g., DataCite DOIs) or guidelines; presence or absence of **citation** metadata can affect **visibility**; may come with administrative tools for central identifier **registration**; besides recording metadata, registration enters identifiers into a database so that the resolver host can look it up and forward requests to the object’s current location; for example, user interfaces and APIs exist for EZID ARKs, DataCite DOIs, Handles, and PURLs

## Application of Identifiers to Newly Collected Field Biocollections

Field biocollections are extraordinarily diverse and continue to grow in scope and scale with the advent of novel technologies such as environmental DNA analyses (e.g., metagenomics), and new continental field-based endeavors such as the National Ecological Observatory Network (NEON; http://www.neoninc.org/) in the United States. Current practices in field collecting are highly heterogeneous and often based on traditional practices of local identifier assignment. Traditionally, “field numbers” are assigned prior to the specimen being fully accessioned. More permanent identifiers (which are also often only locally unique within an institution) are assigned when specimens are accessioned in a collection. In some cases, organizations and communities are already using globally unique identifier systems and even assigning permanent UUIDs for field collection objects while still in the field (as is planned by NEON). In contrast, the geology community has rallied around International Geo Sample Numbers (IGSNs; http://www.geosamples.org/igsnabout), which provide not just global uniqueness, but also minting authority, governance, and a set of services for resolving those numbers that are managed centrally. The lack of consistent practices in biological field sampling compared to what has been accomplished in geology is a lamentable drawback in biodiversity research.

The assignment of local identifiers (e.g., catalog numbers) to specimens for internal management purposes and for external referencing has been the standard practice of biocollections for centuries. As long as humans need to communicate with other humans about specimens, this practice will (and should) continue. By themselves, however, such local identifiers ultimately lead to reduced value of specimens if they are used as the nexus to which all other derived, digitized data connect. The main problem is that local identifiers are not sufficient for linking data across the Internet; globally unique and persistent identifiers are a requirement for this. Thus, to maximize the value of specimens for both human-human communication and human-computer (as well as computer-computer) communication, globally unique identifiers should be issued to data objects together with local identifiers.

### Roadblocks

Providing a chain of provenance for specimens and related data is a major challenge and has a set of roadblocks along multiple dimensions. Traditional field collecting methods are ingrained in many scientists. The informatics community needs to reach out more effectively and explain to scientists the limitations of existing workflows and why an identifier scheme built around global uniqueness is not only necessary from an informatics standpoint, but would dramatically enhance the value of data for re-use, syntheses and analyses. Identifier solutions must support scientists’ current practices and create minimal burden during the collecting process. The solution should provide incentives for adoption, both in the field and in downstream information systems. In particular, effort is needed to ensure perpetuation of field-assigned identifiers through to more permanent data curation steps. Whatever underlying identifier system is chosen, it needs to be robust in preventing the same identifiers from being assigned to different objects (and, ideally, reducing circumstances where the same object receives multiple identifiers).

An additional roadblock is a lack of clarity as to which classes of objects, concepts, or events identifiers should be assigned. Should GUIDs be associated with the actual, physical specimen or with the derived digital (e.g. images) or physical (e.g. tissues) derivatives? Focusing on biocollections specimens as material samples helps semantically clarify what bears the identifier, but many other modeling challenges relating measurement processes etc. to specimens still remain. Even for physical specimens, there are challenges in defining the types of entities that can constitute a specimen, which range from a distinct organism to a part of an organism, to a set of organisms, to abiotic samples containing specimens (e.g., a jar of seawater).

### Next Steps

**Figure 1.**
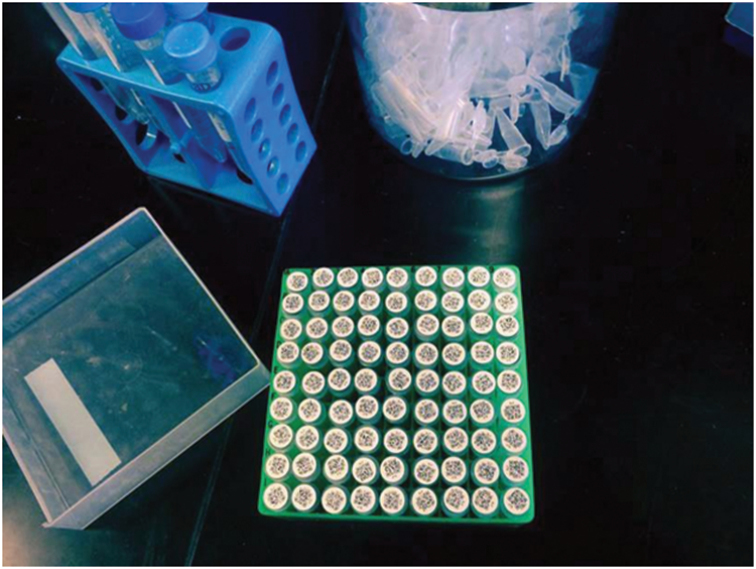
Example of UUIDs embedded within QR-Codes on microcentrifuge tube labels. The 5 mm × 5 mm QR-Codes (Version 2) are printed with a standard laser printer on sheets of self-adhesive 9 mm dots, and scan reliably with a standard barcode reader, while still providing room for a human-readable 5-character prefix + 5-digit number (the human-readable number and UUID are permanently cross-linked in the data management system). Photo: Robert K. Whitton.

**Figure 2.**
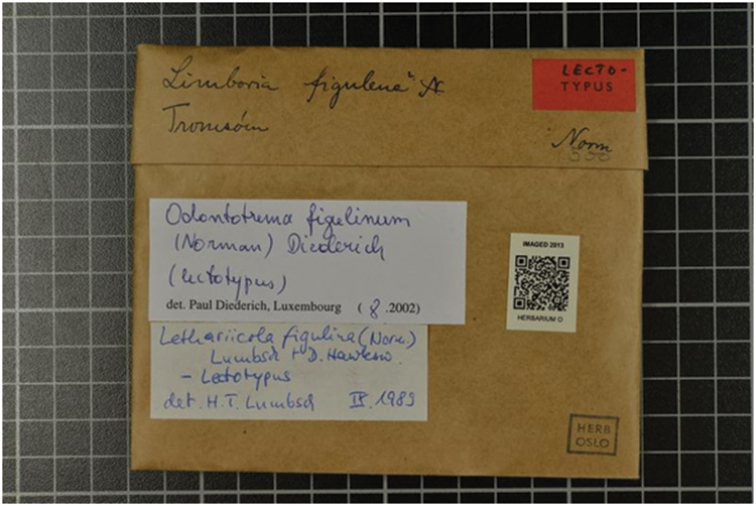
Example of a PURL-URI as a QR-Code, in this example attached to a digitised lichen type specimen in the Natural History Museum, University of Oslo. The QR-Code corresponds to http://purl.org/nhmuio/id/c1a8b878-a4f9-448b-be00-26cbad58b11c.

For newly collected samples, a highly desirable next step is the ability to assign globally unique identifiers directly to newly collected specimens or mixed samples in the field or shortly thereafter. In many cases, it may be desirable that these identifiers be pre-minted and written into a physical barcode or QR-Code, perhaps in conjunction with a human-friendly identifier. Figures 1 and 2 show different examples, the first representing a traditional biocollections object and the second depicting mass-labeling of tubes associated with collections samples. Assigning GUIDs to specimens at the time of collection allows field researchers to publish references to recently collected specimens without waiting for institutional identifiers that are assigned during the accession process. Beyond simply assigning unique identifiers in the field, it is critical that these identifiers persist perpetually with the objects they identify and all descendant samples, subsamples, analyses, data and publications referring to them, ensuring an unbroken chain of data provenance. In the best of all possible worlds, identifiers assigned in the field are retained as the permanent institutional identifier during accessioning.

**Figure 3.**
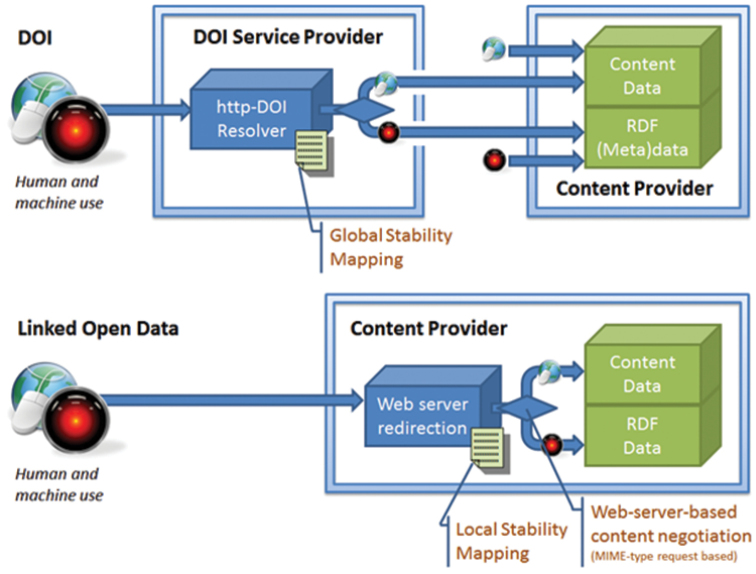
Identifier schemes differ in whether redirections and mappings to ensure stability are centrally managed or not. Top: a DOI dereferencing service like CrossRef or Datacite redirects to the actual content provider; the URIs of content data and RDF metadata are publicly visible and can be used as independent (albeit often unstable) identifiers. Bottom: A linked open data pattern, where each content provider assumes the responsibility for maintaining a stable mapping; the content negotiation is internal. Modified after Hagedorn 2013.

It is not feasible (or, at this stage, even desirable) for the entire biodiversity community to adopt a single implementation for identifiers. However, evaluation of the available technical solutions is a high priority, and the scope of solutions includes IGSNs, DOIs, EZID ARKs, LOD-URIs and UUIDs (comparisons among many of the different options are shown in Table 2 and a comparison of more or less centrally managed mapping and redirection services is shown in Figure 3). The group explored several different viewpoints promoting the utilization of HTTP URIs for all identifiers and did not reach a consensus. HTTP URIs have the advantage that they provide a semantic web compatible default dereferencing method through the standard http protocol and can be flexibly constructed (Hagedorn et al. 2013). The advantage of many identifiers not being a HTTP URI is that the omission of a default dereferencing method avoids potential confusion and may allow for even greater flexibility. However, we recommend all identifiers have the ability to be dereferenceable through at least one http-based service, even if the http-form is not preferred.

**Table 2.** Identifiers schemes according to key characteristics noted in part in Box 2.

Identifier characteristics	DataCite DOI	EZID ARK	OCLC PURL	Self-minted HTTP URI*	LSID	DwC Triplet	UUID
**Globally Unique**	yes	yes	yes	yes	yes	no	yes
**Service Metadata Required for global uniqueness**	yes	yes	yes	yes	yes	no	no
**Per-identifier Cost**	per id or subscription fee	yearly subscription fee	free	free	free	free	free
**Identifier Issuance**	registration	registration **	registration	local	local	local	local
**Human-Friendly**	provider dependent	provider dependent	provider dependent	provider dependent	provider dependent	high	low
**Opacity**	partial	partial	partial	provider dependent	provider dependent	low	high
**Adoption by biodiversity informatics community**	biodiversity publishing	low	low	high	low	collections community	variable
**Adoption by broader informatics infrastructures**	variable	low	variable	high	low	low	high
**Dereferencing Service Integration**	yes	yes	yes	yes	yes	no	no
**Dereferencing Characteristics**							
**Dereferencing Type**	central	central	central	distributed	distributed	N/A	N/A
**Structured Identifier Responses directly from resolver** ***	HTML, RDF/XML	HTML	HTML	provider dependent	yes	N/A	N/A
**Redirection**	yes	yes	yes	possible	possible	N/A	N/A
**Clear Namespace policy and contract**	yes	yes	no	no	no	N/A	N/A
**Resolution service backed by institutions**	yes	yes	no	provider dependent	no	****	****

* Self-minted HTTP URIs may include ARKs or PURLs as well

** ARKs have special mechanisms to extend scalability

*** Structured metadata responses may be available after redirection, depending on the provider (e.g. dublincore.org returns RDF/XML for PURLs)

**** Perhaps, if hosted by a general service (e.g. GrBio for Biocollections, GBIF for occurrence records, etc.)

The group strongly suggested that an immediate next step would be to prototype solutions to create persistent identifiers built on different, existing platforms. Such prototypes would engage stakeholders in testing and feedback in order to refine prototypes. The prototypes could also spawn key actions, including more focused workshops/hackathons, perhaps in the context of the Taxonomic Databases Working Group meetings (TDWG), with the goal of reporting outputs of such trials. TDWG, in particular, is a crucial stakeholder as an international standards organization for biodiversity objects and data.

Scaling up to a larger system will require obtaining funding to support development. A fruitful path would be to align a few organizations that are working nationally or globally (e.g., NEON, iPlant (http://iplant.org), iDigBio, Critical Zone Observatories, Consortium of European Taxonomic Facilities) to adopt an early version of the system and to show interoperability and enhanced ability for tracking specimens and their derivatives as an outcome. For those more at the longer-tail of the specimen curation process, such as smaller biocollections or individual labs, incentives for adopting a system to replace the local numbering systems currently in practice could help coalesce efforts, and could further promote the value of such approaches when putting together data management plans (DMP) for funding agencies. In particular, identifier-specific DMP Tool (https://dmptool.org/) template content should be provided. Finally, with the strong growth of handheld devices, the biodiversity informatics community should work to produce tools for assigning identifiers with such devices.

A more detailed implementation proposal could be specified just for field collections, as part of a TDWG task group, leading to a community input and review process. This would be one key part of a larger effort to identify and reach out to national and international stakeholder groups, including collection managers, aggregators, publishers, scientists, funding agencies, downstream users of the data, and developers of software (e.g., Specify, http://specifyx.specifysoftware.org/; Symbiota, http://symbiota.org/docs/; and in-house software used by aggregators such as GBIF).

## Application of Identifiers to Legacy Data

Legacy specimens can be defined as material already stored in collections. The identifiers being considered here are those referring to collection objects, which may or may not persist in the collections, (e.g., living collections, tissue sample for DNA extraction, ecological specimens). A single physical collection object is a curatorial unit, which may represent only a part of a larger thing (e.g., mammal skeleton, fur, tissues), or may be an aggregate (e.g., lots, fossils with multiple organisms, herbarium sheets with multiple specimens). When aggregates are split (e.g., multiple taxa split into different lots, parasites found on organisms, tissue samples removed), the original identifier generally relates to one of the elements and a new identifier is issued for the additional elements.

Most scientific journals, and even GenBank (http://www.ncbi.nlm.nih.gov/genbank/), make only vague recommendations about citing voucher specimens. The legacy identifier commonly used in the literature for botanical specimens over the last hundreds of years is the collector’s name and collecting/field number, which often represents the collector’s personal series number. The legacy identifier commonly used for zoological specimens is the institution acronym/catalog number. For example, the American Society of Mammalogists makes the following recommendation for the Journal of Mammalogy (http://www.mammalsociety.org/uploads/JM%20Author%20Instructions.pdf):

“All DNA sequences must be submitted to GenBank, and accession numbers provided in the manuscript before publication. Museum catalogue numbers for all voucher specimens (including associated tissue) examined must be included in the manuscript (in an Appendix if numerous).”

### Roadblocks

The single key roadblock with legacy data is the use of local identifiers at all steps during the collection and accessioning process. While these provide means for local provenance tracking, they are insufficient for managing across collections, and are hard to adapt and scale to an open platform for data discovery such as the Internet. A classic example is botanical “duplicates” that come from the same collecting event where different clippings of the same plant were sent to multiple museums. Similar issues can be found for cases where specimens were gifted from one collection to another. In these cases, linkages associating those specimens across collections were typically severed when biocollections were accessioned independently into institutional museum repositories. Those past associations can only be inferred from re-compiling data and looking for content-level matches related to the collections events.

Further, because most collections have effectively developed local curatorial practices, often based on regional and taxon-specific approaches, there is a wide variety of different legacy identifiers associated with specimens and their data. In sum, current practices were and remain highly heterogeneous and the information that could re-associate specimens across collections are lost and cannot be solved simply via post-hoc application of new GUIDs. Thus, the problems with legacy collections are managing both identifiers already in use and dealing with potential application of new ones.

**Next Steps:** As a pragmatic matter, the immediate next steps for legacy collections may not include broad application of globally unique identifiers. Instead, a short term next step is for biodiversity informaticians and collections staff to work together to standardize practices for assigning unique identifiers that are persistent (remain tightly associated with the objects they identify) and stable (continue to be actionable). At a minimum, institutions should clarify the identifier scheme being used locally via their own internal policies. Further development of community-wide best practices would be more effective because they would not only foster local curatorial practice, but also specify how those locally curated materials and their data eventually become part of the rapidly coalescing global, digital framework. These best practices need to be developed in the context of existing efforts and/or organizations such as the Global Registry of Biorepositories (GRBio; http://grbio.org/), which provides a needed framework for publishing repository-specific information like standard acronyms for institutions and collections. Curators should register their collections in GRBio and specify the adopted identifier scheme for the collection.

The legacy group also considered medium-term and longer-term goals, focusing more on broad informatics solutions than local identifier curation practices. One critical step is to assemble identifiers published by curators to aggregators such as GBIF and to assess identifier heterogeneity. This can feed into developing software for comparing identifiers (e.g., resolvers) that is better able to perform fuzzy matching on identifier strings (and fetch such variations), given that identifiers are sometimes expressed in unintended ways (e.g., added spaces or hyphens, capitalization, etc.). Using just a simple string comparison is insufficient and more robust systems should be set in place, which will then forward to the correct identifier. The same applies to whether a URI prefix should be part of the identifier or not.

Next, in order to avoid broken URIs, institution-independent resolvers (e.g., purl.org) or aggregators (e.g., GBIF) should check dereferenceable URIs at certain intervals and inform the responsible contact person when the target URIs return a 404 HTTP status code or are otherwise unavailable. Some providers, such as CrossRef (http://www.crossref.org/), offer services for policing broken URIs. With regards to the data records associated with specimens and published to aggregators such as GBIF, the legacy identifiers group strongly argued for the longer-term goal of inclusion of proper GUIDs in the occurrenceID (or materialSampleID) Darwin Core (Wieczorek et al. 2012) field, rather than some sort of concatenation of local identifiers, such as a Darwin Core triplet (Guralnick et al. 2014). Finally, we strongly encourage integration of identifier metadata into existing standard schemas (e.g., Darwin Core, ABCD; http://www.tdwg.org/activities/abcd/) as new concepts. Such metadata would include information regarding identifiers, persistence, rules for attribution (use, citation, reference) etc.. as is also discussed further in the “cross-cutting solutions” section.

The legacy biocollections group developed a list of immediate action items to most efficiently take the steps listed above. As a priority list, these include:

1. Assemble current identifiers from aggregate data as a means to determine current practices. Some of this work has already been accomplished as part of work by Guralnick et al. (2014) to evaluate Darwin Core Triplets and their current use as identifiers in different systems (e.g., VertNet, http://vertnet.org; Barcode of Life Data Systems, http://www.boldsystems.org/; GenBank), but further work focusing on GBIF datasets is needed. A critical assessment of current implementations will feed into the next step of generating more informed best practices or appropriate strategies that individual institutions can adopt based on their current GUIDs application.
2. Create best practice documentation on known identifier minting schemes. Document best practices with use cases, examples, and pros and cons.
3. As in the new field-collected biocollections group, there is a need to further clarify what exactly is being identified - MaterialSample vs. Organism vs. Occurrence; physical object vs. digital representation.
4. Clearly define the scope of the proposed identifying scheme and what benefits can be gained by it.
5. Demonstrate the implications for publishing in the primary literature.

## Application of Biodiversity Data Identifiers In Publishing

Scientific publications are at the core of science communication and still one of the most powerful means for researchers to share their findings. Biodiversity oriented publications, including historical ones dated from the time of Linnaeus and before, provide one of the most important source of data and information, along with the means to quantitatively assess the impact of biocollections, institutions, and taxonomic groups. This enormous resource ultimately provides needed content for museums worldwide in their efforts to secure continued funding for preserving and digitizing their specimen collections. Although the legacy literature is an essential resource and ultimate home for data derived from biocollections, it remains difficult to mine data from it, and provide the means to cite or track data usage. In the 21st century, these problems magnify as new digital systems are built to support registration of new data and provisioning of older content. By maintaining the currently prevailing model of publishing biodiversity information in formats not readable by machine or not readily harvestable, such as paper or PDF, we further impede efforts to convert data into fluid formats that support new science. One of the solutions to the problem is the wide adoption of identifiers for different data elements normally present in biodiversity publications. We present a set of use cases that would strongly benefit from a system of globally unique identifiers:

1. Use of identifiers for handling data across a registry (e.g., ZooBank), a publisher (e.g., Pensoft; http://www.pensoft.net) and a data aggregator (e.g., Plazi; http://plazi.org), thus providing linkages between all three.
2. Use of DOI identifiers for legacy literature allowing full citations from specimens to formal taxon treatments to other publications and vice versa.
3. Enabling of impact tracking of biological specimens, collections, institutions, and biodiversity data across journal articles.
4. Managing of information about specimens (e.g., occurrence records) in a similar way to publication and citation of data in the scholarly literature. For example, there is no current method to import (e.g., through an API) specimen records from resources such as GBIF into manuscripts, and ensure proper provenance and citations of these.
5. Import and citing of specimen records in publications with their own identifiers generated by the primary data providers or by aggregators (e.g., VertNet, GBIF, iDigBio; http://idigbio.org), paving the way to a wide array of future re-uses, including automated tracking of data usage and impact metrics.
6. Reconciliation of specimen label data with collection records published in literature (e.g., for transcription purposes or usage tracking of collections data) via the identifiers as a needed mechanism for linkage.
7. Aggregation of Web content from biodiversity data contained in publications. For example, articles that benefit from semantic markup allows for parsing and linking of independently published biodiversity data.
8. Use of identifiers to reference needed evidence: “In scholarly literature, whenever and wherever a claim relies upon data, the corresponding data should be cited” (http://www.force11.org/datacitation, principle 3).

### Roadblocks

The difficulties in managing, tracking, and large-scale extracting of citations from any sources other than traditional publications are, in part, due to the paucity of widely adopted, persistent, globally unique and resolvable biodiversity data identifiers. Additionally, extracting specimen, taxon, and other biodiversity data from modern scholarly publications with unstructured formats and little to no markup is needlessly challenging. Another major obstacle is that information about specimens might be published in different places and with different levels of granularity. For example, a specimen might be cited as a holotype in a protologue, then georeferenced and published again in subsequent revisions, perhaps even under a synonym, with images and DNA data appearing separately in other publications. Unless the original specimen collection number is used consistently across all publications, it is difficult, if not impossible, to link together all the important digital derivatives independently generated from that specimen.

A final roadblock is the lack of adoption of advanced publishing approaches, including semantic markup, by almost all publishers in this domain. The TaxPub /Journal Archival Tag Suite provides (Catapano 2010, Penev et al. 2010, 2012) all the necessary functionality and has been successfully implemented by Pensoft in 14 journals, including the registration of the their articles in PubMed and PubMedCentral. However, it places the burden on publishers to adopt new technical approaches that are difficult to meet given a lack of resources and strong incentives for change.

### Next Steps

The key next step is to establish the best practices to generate and assign identifiers as they either propagate from biocollections into the literature or are created during semantically enabled publishing processes. Such practices will assure that publications follow a set of principles ratified by various stakeholders and governments, and perhaps best described broadly in the Force11 data citation principles (https://www.force11.org/datacitation), and more directly for the biodiversity community in the Bouchout Declaration (Bouchout Declaration 2014, http://bouchoutdeclaration.org/). Tools are needed to retrieve identifiers assigned to biological names, taxonomic treatments associated with a name and specimen data discovered in the published records and/or stored in domain specific databases.

Below we summarize critical practices and principles for the use of identifiers in semantically enhanced publications:

1. Publishers should use GUIDs for formally cited or potentially relevant data (e.g., authors, books, articles, taxon names, taxonomic treatments, gene sequences, specimens, etc.) maintained in well- established and widely used external registries.
2. Publishers should issue GUIDs for data first made widely available through document publication (e.g., observation on a species published by an amateur naturalist with no GUID issued by or associated with an Institution).
3. Publishers should provide both human- and machine-readable content (Starr et al. 2015) through resolvable GUIDs for separate elements of an article (e.g., individual images, graphs, tables, supplementary materials, taxonomic treatments, checklists, etc.).
4. Resolvable GUIDs should be used as widely as possible to annotate published content; for example, adding a species to a published checklist should be identified by a GUID, which can be linked to the exact “place” within a published text (e.g., between two species in the checklist).
5. Publishers should use GUIDs and authority files for authors, e.g., ORCID (http://orcid.org/), VIAF (http://viaf.org/), authors of plant names (http://www.kew.org/data/authors.html), ZooBank authors (http://zoobank.org) or internal systems that unambiguously identify names of authors.
6. For the conversion of legacy literature, assign GUIDs to relevant elements that are widely used, resolve to content (e.g., articles, treatments, observation records) and can be a source for Linked Open Data. Whenever possible, use an existing identifier service (such as Plazi for treatments), rather than minting additional identifiers.
7. The identifier system(s) should be sustainable for the long term, highly reliable, and have an API as a backbone service.
8. We note a preference for identifiers used by indexing services (while such services use many kinds of identifiers, CrossRef and DataCite (http://datacite.org) DOIs are the most commonly used). Publishers should link data related to an article and the article itself through their GUIDs (CrossRef and DataCite DOIs cross-referencing service).
9. Identifiers and their metadata related to annotations in publications should be housed and made available by an independent party.

We discussed how systems can be built around identifiers that support all the different participants involved in publishing. Authors are critical participants and should better be able to cite usage of their data from semantically enhanced, rather than unstructured, formats. Publishers can assist authors by making all published data linkable/citable and contributing to specialized databases and/or permanent repositories (e.g. Dryad (http://datadryad.org/) or the Biodiversity Literature Repository (https://zenodo.org/collection/user-biosyslit)). Publishers can also provide authoring tools (such as the Pensoft Writing Tool (PWT) used by the Biodiversity Data Journal – see Smith et al. 2013) that assist authors with entry of structured data (i.e., upfront pre-submission markup and easy data import into the manuscript) to which new or existing identifiers can be assigned or included. Hence, easy data download and export to aggregators from the published paper can be achieved.

To serve the broader community (i.e., beyond authors), publishers can also provide tools to find cited data (e.g., http://refindit.org, which searches across CrossRef, DataCite, Mendeley (http://www.mendeley.com), RefBank (http://refbank.org), Global Names Usage Bank (http://www.globalnames.org/GNUB), Biodiversity Heritage Library (http://www.biodiversitylibrary.org/), Biodiversity Literature Repository (https://zenodo.org/collection/user-biosyslit) and others), as well as an ORCID lookup linked to data creators or owners. Contributing institutions can much more easily assess their institutional impact in biodiversity research output by tracking the usage of identifiers embedded in the articles, as well as better manage intellectual property. For example, publishers can work with organizations such as GRBio to create identifiers for institutions so that all can be cited. Funding agencies can better argue that open access is not only a legal mandate but maximizes their return on investments in terms of products made available to the public. One possible step forward is to create identifiers for funding agencies (e.g., Fundref http://www.crossref.org/fundref/).

## Cross-Cutting Issues and Needs

On the second day of the workshop, a subgroup met to broadly consider cross-cutting issues and needs, given the complexity of semantically interlinked publishing, legacy data, new biocollections, and connections to ecological, biomedical, and climate datasets. The group noted that many needed solutions are described in detail by the Cool URIs W3C Interest Group Note (http://www.w3.org/TR/cooluris/). In addition, the group suggested that promoting any particular approaches and standards apart from W3C efforts should be undertaken as part of the reinvigoration of the TDWG globally unique identifiers task group (http://www.tdwg.org/activities/guid/). Because identifier concerns are cross-cutting and involve research scientists, collectors, curators, publishers, and downstream users, collaboration with additional organizations focused on care of collections, such as the Society for Preservation of Natural History Collections, is needed. Shared responsibility among stakeholders can also break down barriers and enhance knowledge dissemination, helping to bridge the two worlds of physical and digital objects in curation of biocollections.

### Defining the Target of the Identifier

Not all identifier schemes are unambiguous in declaring which identifier refers to an information resource and which to a physical object or abstract concept or event. For instance, an identifier referencing a photo of an eagle on a tree could be identifying the digital photo itself, a photographic print that was later scanned, a reference to the eagle as a physical specimen stored in a museum, the event of capturing the image, or a reference to an individual eagle that exists in nature. Distinguishing concepts such as “digital media”, “print media”, “individual”, and “specimen” is not trivial and ultimately relies on attaching formal descriptions from a biodiversity or biocollections ontology to the identified object. We encourage the use of the Darwin Core Basis Of Record term (http://rs.tdwg.org/dwc/terms/basisOfRecord) to describe the exact nature of the resource. There is a current proposal for tying values for the Basis Of Record term to ontology sources in the Biological Collections Ontology (Walls et al. 2014, Deck et al. in press) which will greatly help in clarifying the concepts underlying identified objects and their downstream use.

### Standardizing Identifier Metadata Requests & Responses

Various identifier schemes behave differently when posting requests and receiving responses; standardized responses are urgently needed. An important example is the standardized content negotiation behavior in the semantic web; other examples are the unified content negotiation by CrossRef and DataCite (http://crosstech.crossref.org/2012/05/crossref_and_datacite_unify_su.html). Identifier metadata can be requested from the service provider not only using Linked Data patterns (which a user cannot do with just a web browser), but also by manipulating the URL endpoint directly, such as URL inflections (https://wiki.ucop.edu/display/Curation/ARK), alternate resolution prefixes, 303 re-directs or hashtags to denote physical objects, or parameter specification in the URL query string. The EZID system provides the ability to deliver DataCite, Dublin Core, CrossRef, or Dublin Core kernel (http://dublincore.org/groups/kernel/spec/) metadata profiles. **A strong recommendation is to create a biodiversity metadata profile to complement these existing profiles.**

### Policy and Contracts

What intention goes into the creation of an identifier, including any contracts and technical specifications? The policies of identifier assigning authority provide information about the expectation of commitment, longevity, use, and re-use. Some identifier schemes require membership and fees in order to create identifiers while others are open and free. Some schemes mandate use of a particular table lookup technology while others do not. Each scheme has its unique history, community, and conditions of use (as described in more detail in Table 2). Whatever method is used for creating the identifier, it **should be publicized explicitly by the identifier authority.** Consumers need to know about the persistence mission of the agency and any potential contracts implied by use of the identifier.

### Persisting GUIDs across Systems

The group discussed issues with contracts about retaining identifiers in downstream systems. **We strongly recommend creating community conventions when re-using data to place special importance on referencing and maintaining earlier identifiers**, especially those with clear policy and behaviour contracts. Use of such conventions provides significant value for data producers and consumers, such as data citation networks, analogous to those produced by CrossRef for journal publications.

### Content Mutability

If a physical object is categorized as “organism” and is later changed to “bulk sample”, does its associated identifier change with it? Does the identifier to a concept change if a spelling error or ambiguous wording is corrected in its definition? Does an information resource identifier change during versioning? Does an identifier guarantee binary identical results, or only identical core-content (which may be embedded in a modified template or formatted differently)? In some cases the answer may be a permanent single mutability policy of the identifier scheme itself, in other cases the identifier scheme may support multiple policies, and the mutability policy may be available as metadata on the identified object. **We recommend using, and where necessary, developing, a vocabulary to document mutability policies and conventions for various content types.**

### Resolver Persistence

Dereferencing, or the automated process that a software tool (e.g., a web browser) employs to go from identifier to content or metadata access, starts with a URL. All identifiers, regardless of scheme, are resolved by a user agent if they are embedded in a URL. As for institutions that have long-term access in their mission, many people think that smaller, newer institutions’ website hostnames will be short-lived compared to those of older, larger institutions (e.g., loc.gov, bnf.fr). Some people prefer to trust a hostname backed by a group of institutions, even if comparatively young (e.g., dx.doi.org), rather than by any one institution. Among such group or consortial arrangements, some people prefer to trust those committed to open access (e.g., gbif.org). Persistence missions can also far exceed current technological solutions. Will, for example, current http protocols look anything like the protocols used in 2065? **Forecasting about resolver persistence for 10, 20, 50, or 100 years is at best educated guesswork, but it should take into account such things as inevitable technological advances, resolver organization’s mission, size, business model, openness, and current age.**

### Identifier Ergonomics and Curation

Identifier readability and ease of transcription are concerns whenever identifiers are routinely recognized, typed, or written by human beings (e.g., on specimen labels). Non-opaque identifiers (containing recognizable strings) tend to be easy to read and to enter because humans can often spot transcription errors; however, it is difficult to mint them uniquely and quickly, and to keep them persistent (their structure makes them prone to “semantic rot”). It is easy to create UUIDs quickly and in large number, which can be especially useful for tracking instances of samples or events in aggregator databases. On the other hand, UUIDs rendered as hexadecimal characters (as opposed to embedded in QR-Codes) are opaque and long, and not as useful in situations where a UUID is expected to be printed onto an insect pin, placed in a vial, or entered via a user interface by hand. There are other means of generating shorter unique opaque identifiers (e.g., Noid), but they have other disadvantages. **One solution to this dilemma is to maintain human-friendly identifiers (e.g., catalog numbers) when presenting content to humans in addition to computer-friendly identifiers (LOD, UUID, DOI, ARK, etc.) for electronic cross-linking.** Such a solution does require curation overhead to assure that both are managed for the long-term. Emerging services such as GRBio maps human-friendly Institution and Collection Codes to URIs for biocollections.

## Conclusions and Planning For The Longer Term

Perhaps the most critical outcome of this workshop was general agreement about a key set of issues, listed below:

1. As opposed to discussing particular implementations, which is likely to be counterproductive, the group was much more interested in cross-cutting issues and the importance of delivery mechanisms that help machines and users interpret identifiers and metadata about them and the biodiversity data objects to which they point.
2. New field-based biocollections and legacy biocollections have different immediate and longer-term needs when it comes to identifier solutions. While there is every reason to assign a globally unique, persistent identifier to new data in biocollections, it may be less critical for legacy records. For legacy data, the problem of broken associations already exists and can only be repaired by spending effort to re-assert the relationships.
3. When a publisher creates records for a new derivative from a legacy collection, it should always copy in the “original” identifier field from the legacy record into the new record. Best practices and conventions for doing so still need to be developed.
4. Publications and data aggregators should not only honor existing identifiers and the metadata about those identifiers, but also follow practices that maximize interoperation with emerging digital library practices regarding data citation.
5. We see great value in reviving or establishing task groups in (and between) TDWG and SPNHC that can help implement some of the best practices and next steps discussed in this document, in particular the creation of a biodiversity metadata profile for identifiers, which can provide critical information about the type of biodiversity object to which the identifier points.

It is noteworthy that the assembled group represented people who have expressed sometimes opposing views on which identifier implementation is most likely to best support sharing and linking biodiversity data. The longer term is likely to see a whole suite of differing solutions, and Table 2 provides more details about differing identifier implementations and services. More important are the cross-cutting solutions, independent of any one identifier implementation, which can best facilitate a vibrant interconnected graph of specimens, samples, images, descriptions/traits, sequences and published content.

## Appendix 1

**Table:** Participants in Identifiers Workshop held October 25–26, 2014 at the Stockholm Museum of Natural History.

Name	Institution/Organization	email
Nico Cellinese	University of Florida	ncellinese@flmnh.ufl.edu
John Deck	University of California, Berkeley	jdeck@berkeley.edu
Rob Guralnick	University of Colorado, Boulder	Robert.Guralnick@colorado.edu
Hilmar Lapp	NESCENT, Duke University	hlapp@nescent.org
Michael Denslow	NEON	mdenslow@neoninc.org
Richard Pyle	Bishop Museum, Honolulu	deepreef@bishopmuseum.org
Donat Agosti	Plazi	agosti@plazi.org
Joan Starr	California Digital Library	Joan.Starr@ucop.edu
Ramona Walls	iPlant Collaborative	rwalls@iplantcollaborative.org
Kerstin Lehnert	IGSN	lehnert@ldeo.columbia.edu
Roderic Page	University of Glasgow	Roderic.Page@glasgow.ac.uk
Karen Cranston	NESCENT	karen.cranston@nescent.org
Terence Catapano	Plazi	catapanoth@gmail.com
John Kunze	California Digital Library	jak@ucop.edu
Markus Döring	GBIF	mdoering@gbif.org
Lyubomir Penev	Pensoft	penev@pensoft.net
Teodor Georgiev	Pensoft	preprint@pensoft.net
John Wieczorek	Museum of Vertebrate Zoology, University of California, Berkeley	tuco@berkeley.edu
Dag Endresen	Natural History Museum, Oslo	dag.endresen@nhm.uio.no
David Schindel	CBOL, Smithsonian	schindeld@si.edu
Greg Riccardi	Florida State University, iDigBio	griccardi@fsu.edu
Deb Paul	Florida State University, iDigBio	dpaul@fsu.edu
David Fichtmueller	Berlin Botanic Garden	d.fichtmueller@bgbm.org
Falko Gloeckler	Natural History Museum, Berlin	falko.gloeckler@mfn-berlin.de
Jana Hoffmann	Natural History Museum, Berlin	jana.hoffmann@mfn-berlin.de
Elspeth Haston	Royal Botanic Garden, Edinburgh	e.haston@rbge.org.uk
